# Healthcare professionals’ knowledge, attitudes, and practices regarding the management of temporomandibular joint disorders: a multicenter, cross-sectional study

**DOI:** 10.1186/s12909-025-08424-9

**Published:** 2025-12-17

**Authors:** Lulu Pan, Hongming Peng, Xi Ding, Xianyu Mao, Pengcheng Ye, Yihui Huang, Bihong Ye, Mengmeng Pan

**Affiliations:** 1 Department of Stomatology, The First Affiliated Hospital of Wenzhou Medical University, Wenzhou, 325027 China; 2 Department of Rehabilitation, Wenzhou TCM Hospital of Zhejiang Chinese Medical University, Wenzhou, 325000 China; 3 Department of Orthodontics, Hospital of Stomatology, Wenzhou Medical University, Wenzhou, 325027 China; 4 Department of Acupuncture and Rehabilitation, Wenzhou TCM Hospital of Zhejiang Chinese Medical University, Wenzhou, 325000 China; 5 Intensive Care Unit, The First Affiliated Hospital of Wenzhou Medical University, Wenzhou, 325027 China

**Keywords:** Temporomandibular joint disorders, Knowledge, attitudes, practice, Healthcare professionals, Cross-Sectional study, Health education

## Abstract

**Background:**

Temporomandibular joint disorders (TMD) are common yet often underdiagnosed conditions requiring appropriate management by healthcare professionals. Assessing their knowledge, attitudes, and practices (KAP) is crucial for improving patient care. This study aims to evaluate healthcare professionals’ knowledge, attitudes, and practices (KAP) related to temporomandibular joint disorders (TMD) and their management strategies.

**Methods:**

This multicenter, cross-sectional survey was conducted on healthcare professionals across hospitals and clinics in Zhejiang Province between November and December 2024. A structured, validated questionnaire was designed to assess demographic characteristics and KAP scores.

**Results:**

Of the 532 valid questionnaires obtained (valid rate: 96.73%), 417 participants (78.38%) were medical doctors. Among respondents, 287 (53.95%) reported prior experience managing temporomandibular joint (TMJ) cases. Mean KAP scores were as follows: knowledge, 22.64 ± 10.11 (possible range: 0–38); attitude, 26.92 ± 3.09 (possible range: 6–30); and practice, 36.97 ± 6.15 (possible range: 9–45). Significant positive correlations were found between knowledge and attitude (*r* = 0.327, *P* < 0.001), knowledge and practice (*r* = 0.455, *P* < 0.001), and attitude and practice (*r* = 0.622, *P* < 0.001). Mediation analysis revealed knowledge directly influenced attitude (β = 0.113, *P* = 0.018) and practice (β = 0.164, *P* = 0.019), while attitude also directly influenced practice (β = 1.064, *P* = 0.007). Additionally, knowledge indirectly affected practice through attitude (β = 0.120, *P* = 0.015).

**Conclusions:**

Healthcare professionals exhibited insufficient knowledge but generally positive attitudes and practices toward TMD management. Targeted educational interventions could significantly improve their knowledge, attitudes, and clinical practices in managing these disorders.

**Clinical trial number:**

not applicable.

**Supplementary Information:**

The online version contains supplementary material available at 10.1186/s12909-025-08424-9.

## Background

Temporomandibular disorders (TMD) are a group of common musculoskeletal conditions predominantly affecting individuals aged 20 to 40, with a higher prevalence among females [1, 2]. The overall prevalence of TMD in the adult population has been reported to range between 27% and 38% [3]. Chronic TMD significantly impairs patients’ ability to work and engage in social interactions, resulting in reduced quality of life [4, 5]. Typical clinical manifestations include pain in the temporomandibular joint region, joint sounds, and restricted mouth opening [6, 7]. These symptoms adversely affect patients’ daily lives, interfering with essential functions such as eating and speaking, and can lead to secondary issues, including sleep disturbances and psychological health problems [8, 9].

The Knowledge, Attitude, and Practice (KAP) theory provides a foundational framework for understanding behavior change. According to this theory, knowledge forms the basis for behavior change, while attitudes and beliefs act as the driving forces behind this process [10]. Behavior change, as outlined in KAP theory, occurs through three sequential stages: acquiring knowledge, developing attitudes or beliefs, and ultimately adopting new practices or behaviors [11]. Notably, knowledge acquisition alone does not guarantee behavior change. It must first result in a shift in perception, which subsequently facilitates changes in behavior through this altered perception [12]. This study holds substantial clinical and educational relevance due to the interdisciplinary nature of TMD diagnosis and treatment, which spans multiple medical specialties, including dentistry, neurology, and otolaryngology. This complexity underscores the importance of healthcare professionals possessing a robust knowledge base and relevant clinical experience. Assessing healthcare providers’ KAP concerning TMD can identify existing gaps in training and inform the development of more effective continuing education strategies.

Furthermore, the diverse symptomatology of TMD often necessitates a multidisciplinary approach to management. Understanding the diagnostic and treatment perspectives of healthcare professionals from various specialties is essential for optimizing care pathways, improving treatment outcomes, and enhancing patient prognoses. Although some studies have investigated the knowledge and attitudes of healthcare professionals regarding TMD, the majority of these focus exclusively on dental practitioners. Examples include surveys of dental postgraduates and practicing dentists in western regions [13] and studies examining the impact of postgraduate education on TMD-related knowledge [14]. However, given the interdisciplinary nature of TMD management, comprehensive research encompassing other relevant specialties remains scarce. Key areas lacking systematic investigation include differences in knowledge and attitudes across various professional backgrounds, collaboration models among disciplines, and variations in treatment strategies. Addressing these gaps is crucial for improving the coordination and efficacy of multidisciplinary TMD care.

Therefore, this study aims to evaluate healthcare professionals’ knowledge, attitudes, and practices (KAP) related to TMD, with a particular focus on their management strategies.

## Methods

### Study design and participants

This multicenter, cross-sectional study was conducted between November and December 2024, on healthcare professionals across hospitals and clinics, including The First Affiliated Hospital of Wenzhou Medical University, Wenzhou Traditional Chinese Medicine Hospital of Zhejiang Chinese Medicine University, the Second Affiliated Hospital of Zhejiang University, the Affiliated Stomatology Hospital of Wenzhou Medical University, Wenzhou Central Hospital, Wenzhou People’s Hospital, Wenzhou Integrated Chinese and Western Medicine Hospital, Wenzhou Taishun County Hospital of Traditional Chinese Medicine and several dental clinics, in Zhejiang Province. The study was approved by the institutional review boards of the First Affiliated Hospital of Wenzhou Medical University. Ethical approval was secured prior to the commencement of the study, and informed consent was obtained from all participants. Participants eligible for inclusion were active healthcare professionals, while those unwilling to participate and students were excluded from the study. I confirm that all methods were performed in accordance with the relevant guidelines. All procedures were performed in accordance with the ethical standards laid down in the 1964 Declaration of Helsinki and its later amendments.

### Questionnaire design

The questionnaire was developed based on established guidelines [15] to ensure content validity. Following its initial drafting, it was revised based on detailed feedback from two subject matter experts, after which a small-scale pilot study involving 51 participants was conducted to evaluate its reliability and feasibility. The internal consistency of the instrument was found to be strong, with a Cronbach’s α of 0.971, confirming its suitability for the study population. The final version, administered in Chinese (a version translated into English was attached as an Appendix), comprised 46 items spanning four dimensions. These included 13 items for demographic information, 9 items assessing knowledge (with several items containing sub-components: item 4 included 4 sub-items; item 5 included 2 sub-items; item 6 included 5 sub-items; and item 9 included 3 sub-items), 6 items evaluating attitudes, and 10 items measuring practices.

The scoring system for the questionnaire was designed to allow detailed assessment of participants’ knowledge, attitudes, and practices. For the knowledge dimension, responses were scored as 2 points for “Well understood,” 1 point for “Heard of,” and 0 points for “Unclear,” resulting in a possible total score range of 0–38. The attitude and practice dimensions both employed a five-point Likert scale, with responses graded from very positive (5 points) to very negative (1 point) based on the positivity of the question. For the attitude dimension, the total possible score range was 6–30, while the practice dimension had a range of 9–45, with item 10 in the latter presented descriptively and excluded from scoring. Scoring definitions were applied to classify participants’ levels across the dimensions: knowledge scores of 0–19 indicated insufficient knowledge, 20–27 represented moderate knowledge, and 28–38 indicated sufficient knowledge. For attitudes, scores of 6–15 were classified as negative, 16–21 as neutral, and 22–30 as positive. Practice scores were categorized as 9–22 for negative behavior, 23–31 for moderate behavior, and 32–45 for positive behavior.

### Questionnaire distribution and quality control

A snowball sampling method was adopted to recruit healthcare professionals through wechat groups and academic forums. An electronic questionnaire was administered using the Sojump platform (http://www.sojump.com), a widely used online survey tool. Participants accessed the questionnaire either by scanning a Quick Response (QR) code or through a link shared in a designated WeChat group. To ensure informed consent, respondents were required to select the option “I agree to participate in this study” before proceeding with the questionnaire. All data were collected anonymously to protect participants’ privacy. To maintain data integrity and prevent duplicate responses, IP address restrictions were implemented, allowing only a single submission from each unique IP address.

### Sample size calculations

Sample size was calculated using the formula for cross-sectional studies [16]: α = 0.05,$\:\:\mathrm{n}={\left(\frac{{Z}_{1-\alpha\:/2}}{\delta\:}\right)}^{2}\times\:p\times\:\left(1-p\right)$ where $\:{Z}_{1-\alpha\:/2}$=1.96 when α = 0.05, the assumed degree of variability of *p*=0.5 maximises the required sample size, and δ is admissible error (which was 5% here). The theoretical sample size was 480 which includes an extra 20% to allow for subjects lost during the study.

### Statistical analysis

Data analysis was conducted using SPSS 27.0 (IBM, Armonk, NY, USA) and AMOS 26.0 (IBM, Armonk, NY, USA). Questionnaire reliability was assessed using Cronbach’s alpha. Descriptive statistics were used to summarize demographic data and KAP (knowledge, attitude, and practice) scores, with continuous variables expressed as mean ± standard deviation (SD) or median (range), depending on normality, and categorical data presented as n (%). Comparisons of KAP scores among demographic groups were performed using independent-samples t-tests for normally distributed data, Mann-Whitney U tests for non-normally distributed data, and one-way analysis of variance (ANOVA) or Kruskal-Wallis tests for comparisons across three or more groups. Spearman correlation analysis was employed to examine correlations among the KAP dimensions, and structural equation modeling (SEM) and mediation analysis using AMOS were conducted to explore the direct or indirect effects among KAP dimensions. A two-sided P-value of less than 0.05 was considered statistically significant.

## Results

### Demographic characteristics

Initially, a total of 550 questionnaires were collected. after data cleaning, 5 cases of questionnaires whose response time was less than 60 s were excluded; 2 cases of questionnaires whose informed consent was selected B were excluded; 11 cases of questionnaires whose KAP dimensions were all selected A were excluded, and the total number of remaining valid questionnaires was 532 (valid rate: 96.73%). Among them, 417 (78.38%) were doctors, 328 (61.65%) were female, 263 (49.44%) were aged 31–40 years, 261 (49.06%) were in third-tier city or below, 377 (70.86%) had Bachelor’s degree, 260 (48.87%) were from Department of Stomatology or Oral and Maxillofacial Surgery, 287 (53.95%) had knowledge related to TMD, 287 (53.95%) had experience in handling TMJ cases, and 181 (34.02%) had followed medical advancements on TMD. The mean knowledge, attitude, and practice scores were 22.64 ± 10.11 (possible range: 0–38), 26.92 ± 3.09 (possible range: 6–30), and 36.97 ± 6.15 (possible range: 9–45), respectively. Analyses of demographic characteristics found that participants’ knowledge, attitude, and practice scores varied across profession (*P* = 0.003, *P* = 0.011, *P* = 0.033), knowledge related to TMD (*P* < 0.001, *P* < 0.001, *P* < 0.001), experience in handling TMD cases (*P* < 0.001, *P* = 0.007, *P* < 0.001), and following medical advancements on TMD (*P* < 0.001, *P* < 0.001, *P* < 0.001). Differences in knowledge scores were also more likely to be found among participants with different gender (*P* = 0.014), education (*P* = 0.021), and department (*P* < 0.001). Furthermore, their practice scores were more likely to vary depending on gender (*P* = 0.021) (Table 1).

**Table 1:** Baseline characteristics

	*N* (%)	Knowledge		Attitude		Practice	
		Mean ± SD	*P*	Mean ± SD	*P*	Mean ± SD	*P*
**Total**	532	22.64 ± 10.11		26.92 ± 3.09		36.97 ± 6.15	
**Profession**			**0.003**		**0.011**		**0.033**
Doctor	417 (78.38)	23.36 ± 9.90		27.10 ± 3.04		37.27 ± 6.28	
Nurse	115 (21.62)	20.03 ± 10.46		26.28 ± 3.21		35.90 ± 5.53	
**Gender**			**0.014**		0.224		**0.021**
Male	204 (38.35)	24.09 ± 10.48		27.20 ± 2.83		37.73 ± 6.24	
Female	328 (61.65)	21.75 ± 9.78		26.75 ± 3.24		36.51 ± 6.05	
**Age**			0.957		0.979		0.362
Below 30 years old	189 (35.53)	22.28 ± 9.78		26.97 ± 2.98		37.03 ± 5.85	
31 ~ 40 years old	263 (49.44)	22.77 ± 10.54		26.90 ± 3.13		37.17 ± 6.40	
Above 40 years old	80 (15.04)	23.11 ± 9.48		26.88 ± 3.24		36.20 ± 6.02	
**City of residence**			0.558		0.651		0.211
First-tier city	51 (9.59)	22.84 ± 10.52		27.35 ± 2.79		38.18 ± 5.75	
Second-tier city	220 (41.35)	23.13 ± 10.51		26.88 ± 3.22		37.15 ± 6.16	
Third-tier city or below	261 (49.06)	22.20 ± 9.69		26.87 ± 3.05		36.59 ± 6.20	
**Education**			**0.021**		0.560		0.182
Associate degree or below	54 (10.15)	22.41 ± 9.72		26.56 ± 3.40		36.85 ± 5.49	
Bachelor’s degree	377 (70.86)	22.05 ± 9.93		26.90 ± 3.05		36.72 ± 6.17	
Master’s degree or above	101 (18.99)	25.01 ± 10.72		27.18 ± 3.09		37.97 ± 6.34	
**Professional title**			0.683		0.094		0.780
None	102 (19.17)	21.37 ± 9.73		26.18 ± 3.33		36.22 ± 6.42	
Junior	185 (34.77)	23.01 ± 9.97		27.17 ± 2.97		37.23 ± 5.95	
Intermediate	178 (33.46)	22.74 ± 10.47		26.97 ± 3.03		37.07 ± 6.23	
Senior(including associate senior)	67 (12.59)	23.33 ± 10.15		27.24 ± 3.09		37.18 ± 6.09	
**Years of work experience**			0.485		0.120		0.526
≤ 5 years	162 (30.45)	22.60 ± 9.99		26.98 ± 3.12		37.42 ± 5.85	
5–10 years	128 (24.06)	23.28 ± 9.98		27.11 ± 2.97		37.04 ± 5.97	
11–15 years	167 (31.39)	21.59 ± 10.32		26.51 ± 3.17		36.39 ± 6.51	
≥ 16 years	75 (14.10)	24.00 ± 10.04		27.40 ± 2.99		37.20 ± 6.25	
**Department**			**< 0.001**		0.989		0.806
Department of Stomatology or Oral and Maxillofacial Surgery	260 (48.87)	26.10 ± 9.15		26.95 ± 3.05		37.08 ± 5.70	
Other department	272 (51.13)	19.34 ± 9.89		26.89 ± 3.14		36.88 ± 6.56	
**Level of hospital**			0.226		0.433		0.943
Tertiary public hospital	310 (58.27)	23.20 ± 10.46		26.87 ± 3.08		37.04 ± 6.09	
Secondary/primary public hospital or community health center	146 (27.44)	22.12 ± 9.18		26.79 ± 3.20		36.78 ± 6.36	
Private hospital	76 (14.29)	21.41 ± 10.34		27.36 ± 2.91		37.08 ± 6.05	
**Teaching hospital**			0.212		0.330		0.870
Yes	321 (60.34)	23.11 ± 10.62		26.81 ± 3.17		37.07 ± 6.14	
No	211 (39.66)	21.94 ± 9.26		27.09 ± 2.97		36.83 ± 6.17	
**Knowledge related to TMD**			**< 0.001**		**< 0.001**		**< 0.001**
Yes	287 (53.95)	26.88 ± 8.97		27.37 ± 2.79		38.60 ± 5.43	
No	245 (46.05)	17.69 ± 9.08		26.39 ± 3.34		35.07 ± 6.40	
**Experience in handling TMD cases**			**< 0.001**		**0.007**		**< 0.001**
Yes	287 (53.95)	26.45 ± 9.44		27.29 ± 2.88		38.06 ± 5.53	
No	245 (46.05)	18.19 ± 9.00		26.49 ± 3.28		35.70 ± 6.59	
**Following medical advancements on TMD**			**< 0.001**		**< 0.001**		**< 0.001**
Yes	181 (34.02)	28.58 ± 9.15		27.55 ± 2.87		39.78 ± 5.01	
No	351 (65.98)	19.58 ± 9.19		26.60 ± 3.16		35.52 ± 6.18	

### Distribution of responses to knowledge, attitude, and practice

The distribution of knowledge dimensions showed that the three questions with the highest number of participants choosing the “Unclear” option were “Temporomandibular Joint Disorder Syndrome needs to be differentiated from the Long Styloid Process.” (K6.5) with 34.02%, “Surgical treatment options for TMD include Arthrocentesis.” (K9.1) with 29.70%, and “Surgical treatment options for TMD include Open Surgery.” (K9.3) with 28.01% (Table S1). Responses to the attitude dimension showed that 39.10% strongly agreed and 35.90% agreed that healthcare professionals do not pay enough attention to temporomandibular joint disorders and their diagnosis and treatment (A6) (Table S2). The distribution of practice dimensions showed that the three questions with the lowest number of participants choosing the “Strongly agree” option were “I keep up with the latest literature on temporomandibular joint disorders and their diagnosis and treatment, and share and discuss it with colleagues.” (P7) with 29.14%, “I participate in or recommend colleagues to attend training and seminars on temporomandibular joint disorders and their diagnosis and treatment.” (P8) with 30.08%, and “I actively participate in or promote the updating of guidelines or expert consensus on temporomandibular joint disorders and their diagnosis and treatment.” (P9) with 30.83% (Table S3). Talking about the sources from which to get information about TMJ disorders and their diagnosis and treatment, the most frequently reported were industry colleagues (32.14%) and internet consultation (28.57%) (Figure S1).

### Correlations among KAP

Further correlation analysis revealed positive correlations between knowledge scores and attitude scores (*r* = 0.327, *P* < 0.001), as well as between knowledge scores and practice scores (*r* = 0.455, *P* < 0.001). Additionally, attitude scores were positively correlated with practice scores (*r* = 0.622, *P* < 0.001) (Table 2).

**Table 2:** Spearman correlation analysis

	Knowledge	Attitude	Practice
Knowledge	1		
Attitude	0.327 (*P* < 0.001)	1	
Practice	0.455 (*P* < 0.001)	0.622 (*P* < 0.001)	1

### SEM analysis

**Fig. 1:**
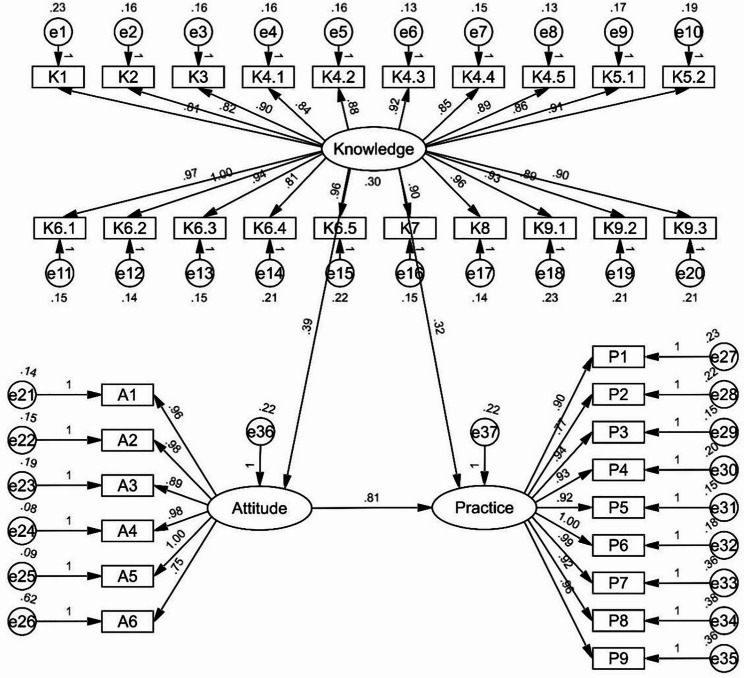
SEM model

The SEM demonstrate a highly favorable model fit indices (CMIN/DF value: 3.663, RMSEA value: 0.071, IFI value: 0.916, TLI value: 0.908, and CFI value: 0.916), suggesting a well-fitting model (Table S4), and the effect estimates between the various paths have been presented (Table 3 and Fig. 1). The results of the mediation analysis based on the SEM shown that knowledge had direct effects on attitude (β = 0.113, *P* = 0.018) and practice (β = 0.164, *P* = 0.019). Meanwhile, attitude had a direct impact on practice (β = 1.064, *P* = 0.007). Furthermore, knowledge indirectly affected practice through attitude (β = 0.120, *P* = 0.015) (Table 4).

**Table 3:** SEM total effect estimates

			β	*P*
Attitude	<---	Knowledge	0.391	< 0.001
Practice	<---	Attitude	0.806	< 0.001
Practice	<---	Knowledge	0.324	< 0.001
K1	<---	Knowledge	0.807	< 0.001
K2	<---	Knowledge	0.823	< 0.001
K3	<---	Knowledge	0.896	< 0.001
K4.1	<---	Knowledge	0.845	< 0.001
K4.2	<---	Knowledge	0.884	< 0.001
K4.3	<---	Knowledge	0.920	< 0.001
K4.4	<---	Knowledge	0.849	< 0.001
K4.5	<---	Knowledge	0.885	< 0.001
K5.1	<---	Knowledge	0.864	< 0.001
K5.2	<---	Knowledge	0.905	< 0.001
K6.4	<---	Knowledge	0.810	< 0.001
K6.5	<---	Knowledge	0.965	< 0.001
K7	<---	Knowledge	0.899	< 0.001
K8	<---	Knowledge	0.965	< 0.001
K6.1	<---	Knowledge	0.967	< 0.001
K6.2	<---	Knowledge	1.000	
K6.3	<---	Knowledge	0.938	< 0.001
K9.3	<---	Knowledge	0.899	< 0.001
K9.2	<---	Knowledge	0.888	< 0.001
K9.1	<---	Knowledge	0.927	< 0.001
A6	<---	Attitude	0.750	< 0.001
A5	<---	Attitude	1.000	
A3	<---	Attitude	0.889	< 0.001
A2	<---	Attitude	0.975	< 0.001
A1	<---	Attitude	0.961	< 0.001
P2	<---	Practice	0.773	< 0.001
P3	<---	Practice	0.942	< 0.001
P4	<---	Practice	0.925	< 0.001
P5	<---	Practice	0.916	< 0.001
P6	<---	Practice	1.000	
P7	<---	Practice	0.992	< 0.001
P8	<---	Practice	0.923	< 0.001
A4	<---	Attitude	0.975	< 0.001
P1	<---	Practice	0.897	< 0.001
P9	<---	Practice	0.964	< 0.001

**Table 4:** Mediation analysis based on the SEM

Model paths			Total effects		Direct Effect		Indirect effect	
			β (95% CI)	*P*	β (95% CI)	*P*	β (95% CI)	*P*
A	<---	K	0.113 (0.088, 0.133)	0.018	0.113 (0.088, 0.133)	0.018	-	-
P	<---	K	0.284 (0.247, 0.321)	0.015	0.164 (0.127, 0.194)	0.019	0.120 (0.096, 0.144)	0.015
P	<---	A	1.064 (0.974, 1.192)	0.007	1.064 (0.974, 1.192)	0.007	-	-

## Discussion

Healthcare professionals demonstrated insufficient knowledge but generally positive attitudes and practices regarding TMD and their management strategies. To enhance the management of TMD, targeted educational interventions are recommended to bridge knowledge gaps and strengthen the link between knowledge, attitudes, and clinical practices among healthcare professionals.

The findings of this study highlight significant gaps in healthcare professionals’ knowledge of TMD, despite generally positive attitudes and practices regarding their management. The disconnect between knowledge and practice is concerning, as previous research has indicated that insufficient knowledge can undermine the effectiveness of clinical decision-making and lead to fragmented care [17, 18]. Although participants demonstrated a strong belief in the importance of addressing TMD and educating patients, their limited understanding of diagnostic differentiation, etiological factors, and treatment pathways suggests a lack of exposure to specialized training. This lack of focus on niche areas has broader implications, as inadequate training in such conditions may prevent healthcare professionals from delivering evidence-based, comprehensive care. Moreover, emerging evidence further illustrates the complexity of TMD etiology. For example, studies have suggested that habitual unilateral mastication is associated with an increased risk of ipsilateral TMD signs and symptoms [19, 20]. These findings support the view that functional loading patterns may contribute to TMD development and reinforce that debates concerning etiological mechanisms and the role of etiological treatment remain ongoing.

These deficiencies are further contextualized by reliance on informal sources of information, as reflected in the preference for internet consultations and industry colleagues over academic resources. Moreover, the absence of universally accepted etiological diagnostic criteria for TMDs further contributes to inconsistencies in clinical understanding and may limit the development of standardized training and management strategies. Similar patterns have been observed in other healthcare studies, where professionals cite time constraints and limited access to academic databases as barriers to engaging with peer-reviewed literature [21, 22]. The observed reliance on informal information sources further emphasizes the need for accessible, high-quality academic resources. The low engagement with peer-reviewed literature and evidence-based guidelines reflects broader challenges in professional education and resource distribution. This dependence on non-academic sources could perpetuate outdated or incomplete knowledge, ultimately hindering the adoption of best practices. In addition, further research aimed at clarifying the etiological mechanisms of TMDs remains essential for supporting the development of reliable diagnostic tools and evidence-based therapeutic approaches.

The correlation and mediation analyses provide important insights into the interplay between knowledge, attitudes, and practices. Knowledge was found to influence practices both directly and indirectly through attitudes, underscoring its foundational role in shaping clinical behavior. However, the stronger relationship between attitudes and practices suggests that positive perceptions alone may drive certain behaviors, even in the absence of comprehensive knowledge. One possible explanation is that self-reported attitudes and practices may be influenced by professional bias or perceived competence, leading participants to rate their behaviors more positively than their actual level of knowledge would suggest. Such tendencies have also been noted in other healthcare contexts, where clinicians report favorable attitudes toward clinical decision-making or patient-centered care despite limited foundational knowledge, often due to perceived expectations or confidence derived from routine clinical experience [17, 18]. In addition, studies examining continuing education and information-seeking behaviors in healthcare professionals highlight that time constraints, reliance on informal sources, and inconsistent access to academic literature may further reinforce an inflated sense of competence, contributing to discrepancies between objective knowledge and self-reported behaviors [21, 22]. These findings support the possibility that similar mechanisms may have contributed to the comparatively high attitude and practice scores observed in our study. Nonetheless, without adequate foundational understanding, healthcare professionals may struggle to adapt to complex clinical scenarios or integrate new advancements into their practice. For example, the low engagement with activities such as updating clinical guidelines or participating in professional training reflects systemic challenges, including insufficient institutional support and resource allocation, which have also been documented in similar healthcare contexts [23, 24].

The detailed analysis of knowledge, attitudes, and practices reveals specific areas that require targeted interventions. Healthcare professionals’ limited familiarity with advanced diagnostic techniques, such as imaging and arthroscopy, indicates gaps in both undergraduate and postgraduate education. Similar deficiencies have been reported in other specialties, where the lack of hands-on training and exposure to advanced diagnostic tools limits clinicians’ ability to provide accurate diagnoses [25, 26]. Furthermore, while attitudes toward TMD management were generally positive, discrepancies in practices—such as limited participation in guideline development and low uptake of professional development opportunities—highlight the impact of broader systemic issues. Similar findings have been reported in other regions, where studies from India, Italy, Pakistan, and other settings have also documented limited knowledge of TMD etiology, diagnosis, and management among dental practitioners, despite generally positive attitudes or established treatment preferences [27–30]. Studies have shown that organizational structures and resource availability significantly influence professional behaviors, particularly in resource-limited settings where continuing education is not prioritized [31, 32].

Addressing these gaps requires a multifaceted approach. First, systemic reforms should prioritize the integration of TMD-specific modules into medical curricula at both undergraduate and postgraduate levels. These modules should focus on enhancing diagnostic skills, understanding etiological complexity, and developing evidence-based treatment plans. Additionally, establishing mandatory continuing education programs on TMD, supported by government or professional bodies, could ensure consistent knowledge updates. Evidence from similar initiatives in other healthcare fields suggests that structured, incentivized training programs significantly improve knowledge retention and application [33, 34]. Second, healthcare organizations should invest in creating accessible, evidence-based resources, such as interactive online platforms or mobile applications, to facilitate self-directed learning. These resources could bridge the gap for professionals who rely on informal sources of information due to time constraints or limited access to academic materials.

Professional collaboration should also be encouraged through interdisciplinary forums or networks, allowing for knowledge exchange and the development of best practices. Moreover, healthcare institutions should provide protected time for professional development activities, ensuring that clinicians can engage in workshops, seminars, or guideline updates without compromising their clinical responsibilities [35, 36]. These initiatives could be further supported by integrating TMD management into broader healthcare strategies, emphasizing its importance as a component of overall patient care.

Long-term sustainability of these reforms requires a coordinated effort among stakeholders, including healthcare professionals, institutional leaders, policymakers, and educational bodies [37, 38]. Allocating resources to support these initiatives, such as funding for training programs and infrastructure development, is essential. Additionally, monitoring and evaluation mechanisms should be implemented to assess the effectiveness of these interventions and guide future improvements [39]. By addressing both systemic and individual-level factors, these strategies have the potential to improve not only knowledge but also attitudes and practices, ultimately enhancing the quality of care provided to patients with TMD.

This study has several limitations that should be acknowledged. First, as a cross-sectional survey, it captures data at a single time point, limiting the ability to infer causality between knowledge, attitudes, and practices. Second, the reliance on self-reported data may introduce response bias, as participants might overestimate their knowledge or practices to align with perceived expectations. Third, the study was conducted in a single province, which may restrict the generalizability of the findings to healthcare professionals in other regions or healthcare systems. Finally, Snowball sampling may introduce selection bias because participants tend to recruit colleagues within their own professional or social networks, resulting in a non-random and potentially homogeneous sample in which individuals do not have an equal probability of being included.

## Conclusion

In conclusion, healthcare professionals demonstrated insufficient knowledge but generally positive attitudes and practices regarding TMD and its management strategies, with knowledge significantly influencing both attitudes and practices directly and indirectly. Targeted educational initiatives and training programs should be implemented to enhance healthcare professionals’ knowledge, thereby improving their attitudes and practices and ultimately optimizing TMD management in clinical settings.

## Supplementary Information

Supplementary Material 1.

Supplementary Material 2.

Supplementary Material 3.

Supplementary Material 4.

## Data Availability

All data generated or analysed during this study are included in this published article.

## Abbreviations

TMD: Temporomandibular joint disorders
KAP: Knowledge, attitudes, and practices
TMJ: Temporomandibular joint
SD: Standard deviation
ANOVA: Analysis of variance
SEM: Structural equation modeling
