# Supplementary figures and images for: Healthcare professionals’ knowledge, attitudes, and practices regarding the management of temporomandibular joint disorders: a multicenter, cross-sectional study
Source: BMC Med Educ. 2025 Dec 17;26:111. doi: 10.1186/s12909-025-08424-9 (PMC12821944; doi:10.1186/s12909-025-08424-9)

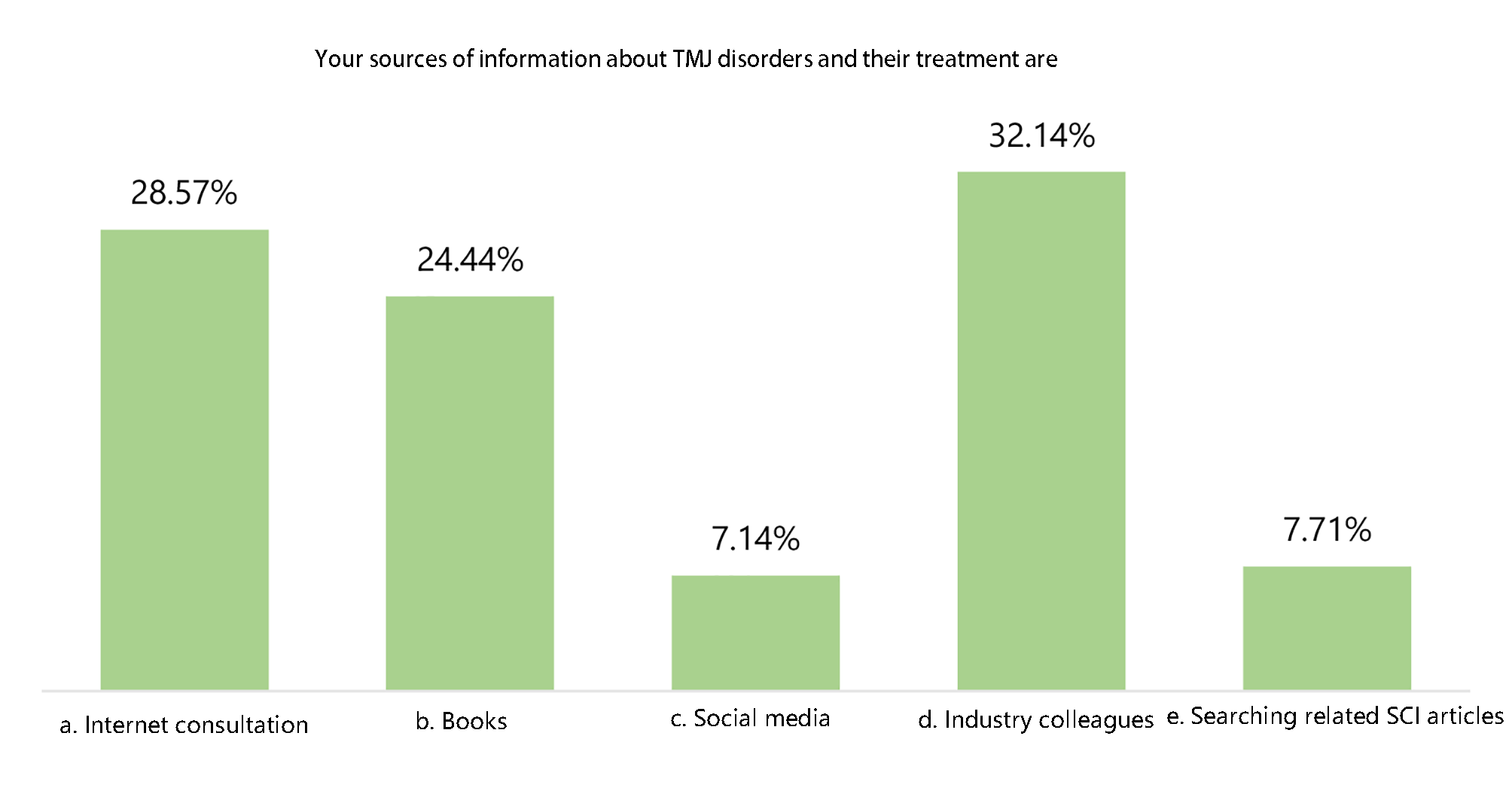

Supplement: Supplementary file 1 — Supplementary Material 1. [file 12909_2025_8424_MOESM1_ESM.tif]
