# Supplementary material for: Healthcare professionals’ knowledge, attitudes, and practices regarding the management of temporomandibular joint disorders: a multicenter, cross-sectional study
Source: BMC Med Educ. 2025 Dec 17;26:111. doi: 10.1186/s12909-025-08424-9 (PMC12821944; doi:10.1186/s12909-025-08424-9)
Supplement: Supplementary file 2 — Supplementary Material 2. [file 12909_2025_8424_MOESM2_ESM.docx]

**Supplementary Materials**

**Figure S1. The sources from which you obtain information about temporomandibular joint disorders and their diagnosis and treatment**

a. Internet consultation

b. Books

c. Social media

d. Industry colleagues

e. Searching related SCI articles
