# Supplementary material for: Healthcare professionals’ knowledge, attitudes, and practices regarding the management of temporomandibular joint disorders: a multicenter, cross-sectional study
Source: BMC Med Educ. 2025 Dec 17;26:111. doi: 10.1186/s12909-025-08424-9 (PMC12821944; doi:10.1186/s12909-025-08424-9)
Supplement: Supplementary file 3 — Supplementary Material 3. [file 12909_2025_8424_MOESM3_ESM.docx]

**Table S1. Distribution of responses to knowledge dimension**

|  | **Very familiar** | **Heard of it** | **Unclear** |
| --- | --- | --- | --- |
| 1. Temporomandibular disorders (TMD) are common conditions of the maxillofacial region, frequently observed in women aged 20–40. | 206 (38.72) | 267 (50.19) | 59 (11.09) |
| 2. Primary symptoms of TMD include:1）Pain or joint clicking when opening or closing the mouth 2）Jaw muscle pain upon waking, often due to teeth grinding 3）Difficulty opening the mouth wide 4）Frequent headaches or neck pain 5）Pain in the temporomandibular joint (TMJ) area 6）"Locking" of the joint 7）Muscle pain or tenderness, restricted jaw movement, and occasionally pain extending to one side of the face. | 239 (44.92) | 254 (47.74) | 39 (7.33) |
| 3. TMD is associated with multiple factors, such as internal or external joint trauma, psychological factors (e.g., depression, anxiety, irritability, stress), malocclusion, autoimmune issues, excessive TMJ load, anatomical abnormalities, and poor habits. | 222 (41.73) | 261 (49.06) | 49 (9.21) |
| 4. Different types of TMD are associated with specific causes: |  |  |  |
| 4.1Masticatory Muscle Disorders: Trauma, mental stress, cold stimulation, nocturnal bruxism, etc., can lead to direct damage to the masticatory muscles. Excessive mouth opening or prolonged mouth opening due to dental treatment, etc., can lead to overactivity of the masticatory muscles, eventually resulting in muscle fatigue. | 216 (40.60) | 274 (51.5) | 42 (7.89) |
| 4.2. Joint Structural Disorders: Temporomandibular joint structural disorders include various types of disc displacement. The etiology is unclear, but many scholars believe it is related to abnormal stress, such as sudden biting of hard objects, sudden excessive mouth opening, trauma, etc., which can cause rapid or excessive movement of the condyle, leading to stretching or tearing of the disc and its attached ligaments, resulting in disc displacement. | 210 (39.47) | 273 (51.32) | 49 (9.21) |
| 4.3. Inflammatory Diseases: Temporomandibular joint synovitis can be classified into primary and secondary types. The etiology of primary synovitis is unclear. Secondary synovitis is often caused by factors such as trauma, inflammation of adjacent joint tissues, or disc displacement, leading to sterile inflammation. | 164 (30.83) | 309 (58.08) | 59 (11.09) |
| 4.3. Osteoarthritis: Osteoarthritis can also be classified into primary and secondary types. The etiology of primary osteoarthritis is unclear, but some scholars believe it is related to mechanical damage, chemical inflammation, and other factors. | 161 (30.26) | 311 (58.46) | 60 (11.28) |
| 4.4. Secondary Osteoarthritis: Primarily caused by local factors such as disc displacement, continuous abnormal pressure on the joint, biting hard objects, trauma, etc. During this process, excessive external force on the mandibular condyle damages the articular cartilage and subchondral bone, leading to the development of osteoarthritis. | 156 (29.32) | 318 (59.77) | 58 (10.90) |
| 5. Diagnostic tests for TMD include: |  |  |  |
| 5.1. Imaging tests: X-rays, cone-beam computed tomography (CBCT), magnetic resonance imaging (MRI), or arthrography. | 214 (40.23) | 270 (50.75) | 48 (9.02) |
| 5.2. Specialized tests: TMJ arthroscopy and dental arch models. | 119 (22.37) | 301 (56.58) | 112 (21.05) |
| 6. Temporomandibular Joint Disorder Syndrome Needs to Be Differentiated from the Following Diseases: |  |  |  |
| 6.1. Maxillofacial Tumors: Tumors in the temporomandibular joint area, the temporomandibular fossa, the posterior wall of the maxillary sinus, the parotid gland area, and the nasopharyngeal area can also cause tooth clenching or difficulty opening the mouth. This may be confused with temporomandibular joint disorder. Differentiation can be made by the presence of neurological symptoms or other symptoms, as well as CT, MRI, and other imaging examinations. | 144 (27.07) | 296 (55.64) | 92 (17.29) |
| 6.2. Temporomandibular Joint Arthritis: 1）Acute Suppurative Temporomandibular Joint Arthritis: Redness, swelling, and significant tenderness in the temporomandibular joint area, especially with an inability to bite the upper and lower teeth together. Pain in the joint area is triggered by slight pressure. 2）Rheumatoid Temporomandibular Joint Arthritis: Characterized by systemic migratory polyarthritis, especially involving small joints of the limbs. In the late stage, joint ankylosis can occur. Differentiation can be made through immunological tests and clinical presentation. | 120 (22.56) | 298 (56.02) | 114 (21.43) |
| 6.3. Ear-Related Diseases: Pain caused by otitis media can radiate to the joint area and affect opening and chewing functions. Differentiation can be made by ear examination. | 122 (22.93) | 309 (58.08) | 101 (18.98) |
| 6.4. Cervical Spondylosis: Can cause pain in the neck, shoulder, back, ear area, and face, which is often misdiagnosed. However, this pain is not related to mouth opening or chewing, but is often related to posture and neck movement, sometimes accompanied by sensory and motor abnormalities in the hands. Differentiation can be made by symptoms and imaging examinations. | 122 (22.93) | 314 (59.02) | 96 (18.05) |
| 6.5. Long Styloid Process: The styloid process of the temporal bone is a thin, long bony protrusion extending forward and downward from the temporal bone. The normal length is about 2.5 cm, and anything longer than 3.5 cm is considered a long styloid process. This condition can cause pain behind the condyle during mouth opening and chewing, as well as referred pain in the joint posterior, ear, and neck areas. Imaging examinations can confirm the diagnosis. | 98 (18.42) | 253 (47.56) | 181 (34.02) |
| 7. Some TMD symptoms may resolve or improve through self-care and lifestyle changes. Others require further treatment. | 178 (33.46) | 297 (55.83) | 57 (10.71) |
| 8. Surgery is not always the next step when conservative treatments fail. For joint structure issues, surgery might help, but it is ineffective for muscle-related problems. | 148 (27.82) | 302 (56.77) | 82 (15.41) |
| 9. Surgical treatment options for TMD include: |  |  |  |
| 9.1. Arthrocentesis: Generally performed in an outpatient setting under local anesthesia in the joint area. A puncture needle is inserted into the joint cavity, and 20–50 ml of saline solution is used for closed, pressurized repeated irrigation, or 200 ml for open continuous irrigation. | 106 (19.92) | 268 (50.38) | 158 (29.70) |
| 9.2. Arthroscopy: A specialized small arthroscope is used to enter the temporomandibular joint, allowing the surgeon to observe its interior and perform minor procedures such as repositioning the joint disc, repairing damaged cartilage, or injecting medication under the scope. | 105 (19.74) | 292 (54.89) | 135 (25.38) |
| 9.3 Open Surgery: An incision is made in front of the ear to expose the joint for the repair of the joint disc or ligaments. In cases of severe joint damage, more complex surgical procedures may be required to address the problem, such as bone reshaping, joint disc replacement, and in some cases, total joint replacement surgery. | 97 (18.23) | 286 (53.76) | 149 (28.01) |

Table S2 **Distribution of responses to attitude** dimension

|  | **Strongly agree** | **Agree** | **Neutral** | **Disagree** | **Strongly disagree** |
| --- | --- | --- | --- | --- | --- |
| 1. I believe that temporomandibular joint disorders are a health issue that patients must pay attention to. | 338 (63.53) | 160 (30.08) | 33 (6.20) | 0 | 1 (0.19) |
| 2. I believe that healthcare professionals should play an important role in guiding the diagnosis and treatment of temporomandibular joint disorder patients. | 322 (60.53) | 176 (33.08) | 32 (6.02) | 1 (0.19) | 1 (0.19) |
| 3. I believe that patients with temporomandibular joint disorders need further examination to determine the treatment plan. | 332 (62.41) | 168 (31.58) | 30 (5.64) | 0 | 2 (0.38) |
| 4. I believe that education on temporomandibular joint disorders and their diagnosis and treatment should be increased for patients. | 351 (65.98) | 157 (29.51) | 23 (4.32) | 1 (0.19) | 0 |
| 5. I believe that education and training on temporomandibular joint disorders and their diagnosis and treatment should be increased for healthcare professionals in relevant departments. | 323 (60.71) | 179 (33.65) | 30 (5.64) | 0 | 0 |
| 6. I believe that healthcare professionals do not pay enough attention to temporomandibular joint disorders and their diagnosis and treatment. | 208 (39.10) | 191 (35.90) | 113 (21.24) | 17 (3.20) | 3 (0.56) |

**Table S3 Distribution of responses to practice dimension**

|  | **Strongly agree** | **Agree** | **Neutral** | **Disagree** | **Strongly disagree** |
| --- | --- | --- | --- | --- | --- |
| 1. I actively seek to understand knowledge related to temporomandibular joint disorders. | 196 (36.84) | 206 (38.72) | 126 (23.68) | 4 (0.75) | 0 |
| 2. I recommend that patients undergo necessary examinations to determine subsequent treatment plans. | 232 (43.61) | 229 (43.05) | 67 (12.59) | 3 (0.56) | 1 (0.19) |
| 3. I educate patients on how to recognize the symptoms of temporomandibular joint disorders and take appropriate self-management measures. | 225 (42.29) | 224 (42.11) | 72 (13.53) | 10 (1.88) | 1 (0.19) |
| 4. I guide patients in practicing relief techniques for temporomandibular joint disorders, such as heat application and relaxation techniques. | 228 (42.86) | 224 (42.11) | 66 (12.41) | 11 (2.07) | 3 (0.56) |
| 5. I provide patients with advice on the prevention of temporomandibular joint disorders. | 228 (42.86) | 223 (41.92) | 72 (13.53) | 9 (1.69) | 0 |
| 6. I take the time to explain in detail the possible causes and treatment options for temporomandibular joint disorders. | 193 (36.28) | 214 (40.23) | 110 (20.68) | 15 (2.82) | 0 |
| 7. I keep up with the latest literature on temporomandibular joint disorders and their diagnosis and treatment, and share and discuss it with colleagues. | 155 (29.14) | 180 (33.83) | 165 (31.02) | 30 (5.64) | 2 (0.38) |
| 8. I participate in or recommend colleagues to attend training and seminars on temporomandibular joint disorders and their diagnosis and treatment. | 160 (30.08) | 203 (38.16) | 142 (26.69) | 23 (4.32) | 4 (0.75) |
| 9. I actively participate in or promote the updating of guidelines or expert consensus on temporomandibular joint disorders and their diagnosis and treatment. | 164 (30.83) | 203 (38.16) | 131 (24.62) | 33 (6.20) | 1 (0.19) |

**Table S4. SEM fit indicators**

| **Indicators** | **Reference** | **Actual** |
| --- | --- | --- |
| CMIN/DF | 1-3: Excellent, 3-5: Good | 3.663 |
| RMSEA | <0.08: Good | 0.071 |
| IFI | >0.8: Good | 0.916 |
| TLI | >0.8: Good | 0.908 |
| CFI | >0.8: Good | 0.916 |
