# Supplementary material for: Healthcare professionals’ knowledge, attitudes, and practices regarding the management of temporomandibular joint disorders: a multicenter, cross-sectional study
Source: BMC Med Educ. 2025 Dec 17;26:111. doi: 10.1186/s12909-025-08424-9 (PMC12821944; doi:10.1186/s12909-025-08424-9)
Supplement: Supplementary file 4 — Supplementary Material 4. [file 12909_2025_8424_MOESM4_ESM.docx]

| Questionnaire ID： |
| --- |
| Dear Participant：  We are researchers from the First Affiliated Hospital of Wenzhou Medical University, and we sincerely invite you to participate in our study. This research aims to understand healthcare professionals' knowledge, attitudes, and practices regarding temporomandibular joint disorders (TMD) and its treatment, providing a basis for developing scientific intervention strategies. These efforts may help improve the health of more people in the future. Your participation is voluntary, and this study has been approved by the Ethics Review Committee. If you agree to participate, please read the following instructions:  1. Please complete the questionnaire. There are no right or wrong answers; simply respond based on your actual situation. If you have any questions during the process, feel free to contact us. Once completed, please submit the questionnaire promptly.  2. This study involves a simple questionnaire survey that will not cause harm to your physical or mental health. However, it does include some personal questions, such as your gender and age. We will strictly maintain confidentiality and ensure your information is not disclosed. Please feel assured while answering.  3. As a participant, you are entitled to learn about the research-related information and progress at any time. If you decide to withdraw from the study, please inform us, and your data will not be included in the research results.  Finally, we sincerely thank you for taking the time to support our scientific research!  □I have been informed and agree to allow the collected data to be used for scientific research.  Signature of Informed Consent：  Date of Participation**:** _____ Year _____ Month _____ Day |

| **Part 1 Basic Information** | |
| --- | --- |
| 1. Profession | a. Doctor  b. Nurse |
| 2. Your gender | a. Male  b. Female |
| 3. Age | a. Below 20 years old b. 21–30 years old  c. 31–40 years old d. 41–50 years old e. Above 50 years old |
| 4. City of residence | a. First-tier city b. Second-tier city c. Third-tier city or below |
| 5. Education level | a. Associate degree or below b. Bachelor’s degree c. Master’s degree d. Doctoral degree or above |
| 6. Professional title | a. None b. Junior c. Intermediate d. Senior (including Associate Senior) |
| 7. Years of work experience | a. ≤5 years b. 5–10 years c. 11–15 years d. ≥16 years |
| 8. Department | a. Department of Stomatology or Oral and Maxillofacial Surgery b. Other departments |
| 9. The level of your hospital | a. Tertiary public hospital b. Secondary/primary public hospital or community health center c. Private hospital |
| 10. Is your hospital a teaching hospital? | a. Yes  b. No |
| 11. Have you ever participated in lectures, presentations, or training related to TMD? | a. Yes  b. No |
| 12. Have you ever had experience handling TMD cases? | a. Yes  b. No |
| 13. Do you regularly follow the latest medical advancements and literature on TMD? | a. Yes  b. No |

| **Part 2 Knowledge of Temporomandibular Disorders and Their Management** | | | |
| --- | --- | --- | --- |
| 1. Temporomandibular disorders (TMD) are common conditions of the maxillofacial region, frequently observed in women aged 20–40. | a. very familiar | d. heard of it | e. unclear |
| 2. Primary symptoms of TMD include:1）Pain or joint clicking when opening or closing the mouth 2）Jaw muscle pain upon waking, often due to teeth grinding 3）Difficulty opening the mouth wide 4）Frequent headaches or neck pain 5）Pain in the temporomandibular joint (TMJ) area 6）"Locking" of the joint 7）Muscle pain or tenderness, restricted jaw movement, and occasionally pain extending to one side of the face. | a. very familiar | d. heard of it | e. unclear |
| 3. TMD is associated with multiple factors, such as internal or external joint trauma, psychological factors (e.g., depression, anxiety, irritability, stress), malocclusion, autoimmune issues, excessive TMJ load, anatomical abnormalities, and poor habits. | a. very familiar | d. heard of it | e. unclear |
| 4. Different types of TMD are associated with specific causes: |  |  |  |
| 4.1Masticatory Muscle Disorders: Trauma, mental stress, cold stimulation, nocturnal bruxism, etc., can lead to direct damage to the masticatory muscles. Excessive mouth opening or prolonged mouth opening due to dental treatment, etc., can lead to overactivity of the masticatory muscles, eventually resulting in muscle fatigue. | a. very familiar | d. heard of it | e. unclear |
| 4.2. Joint Structural Disorders: Temporomandibular joint structural disorders include various types of disc displacement. The etiology is unclear, but many scholars believe it is related to abnormal stress, such as sudden biting of hard objects, sudden excessive mouth opening, trauma, etc., which can cause rapid or excessive movement of the condyle, leading to stretching or tearing of the disc and its attached ligaments, resulting in disc displacement. | a. very familiar | d. heard of it | e. unclear |
| 4.3. Inflammatory Diseases: Temporomandibular joint synovitis can be classified into primary and secondary types. The etiology of primary synovitis is unclear. Secondary synovitis is often caused by factors such as trauma, inflammation of adjacent joint tissues, or disc displacement, leading to sterile inflammation. | a. very familiar | d. heard of it | e. unclear |
| 4.3. Osteoarthritis: Osteoarthritis can also be classified into primary and secondary types. The etiology of primary osteoarthritis is unclear, but some scholars believe it is related to mechanical damage, chemical inflammation, and other factors. | a. very familiar | d. heard of it | e. unclear |
| 4.4. Secondary Osteoarthritis: Primarily caused by local factors such as disc displacement, continuous abnormal pressure on the joint, biting hard objects, trauma, etc. During this process, excessive external force on the mandibular condyle damages the articular cartilage and subchondral bone, leading to the development of osteoarthritis. | a. very familiar | d. heard of it | e. unclear |
| 5. Diagnostic tests for TMD include: |  |  |  |
| 5.1. Imaging tests: X-rays, cone-beam computed tomography (CBCT), magnetic resonance imaging (MRI), or arthrography. | a. very familiar | d. heard of it | e. unclear |
| 5.2. Specialized tests: TMJ arthroscopy and dental arch models. | a. very familiar | d. heard of it | e. unclear |
| 6. Temporomandibular Joint Disorder Syndrome Needs to Be Differentiated from the Following Diseases: |  |  |  |
| 6.1. Maxillofacial Tumors: Tumors in the temporomandibular joint area, the temporomandibular fossa, the posterior wall of the maxillary sinus, the parotid gland area, and the nasopharyngeal area can also cause tooth clenching or difficulty opening the mouth. This may be confused with temporomandibular joint disorder. Differentiation can be made by the presence of neurological symptoms or other symptoms, as well as CT, MRI, and other imaging examinations. | a. very familiar | d. heard of it | e. unclear |
| 6.2. Temporomandibular Joint Arthritis: 1）Acute Suppurative Temporomandibular Joint Arthritis: Redness, swelling, and significant tenderness in the temporomandibular joint area, especially with an inability to bite the upper and lower teeth together. Pain in the joint area is triggered by slight pressure. 2）Rheumatoid Temporomandibular Joint Arthritis: Characterized by systemic migratory polyarthritis, especially involving small joints of the limbs. In the late stage, joint ankylosis can occur. Differentiation can be made through immunological tests and clinical presentation. | a. very familiar | d. heard of it | e. unclear |
| 6.3. Ear-Related Diseases: Pain caused by otitis media can radiate to the joint area and affect opening and chewing functions. Differentiation can be made by ear examination. | a. very familiar | d. heard of it | e. unclear |
| 6.4. Cervical Spondylosis: Can cause pain in the neck, shoulder, back, ear area, and face, which is often misdiagnosed. However, this pain is not related to mouth opening or chewing, but is often related to posture and neck movement, sometimes accompanied by sensory and motor abnormalities in the hands. Differentiation can be made by symptoms and imaging examinations. | a. very familiar | d. heard of it | e. unclear |
| 6.5. Long Styloid Process: The styloid process of the temporal bone is a thin, long bony protrusion extending forward and downward from the temporal bone. The normal length is about 2.5 cm, and anything longer than 3.5 cm is considered a long styloid process. This condition can cause pain behind the condyle during mouth opening and chewing, as well as referred pain in the joint posterior, ear, and neck areas. Imaging examinations can confirm the diagnosis. | a. very familiar | d. heard of it | e. unclear |
| 7. Some TMD symptoms may resolve or improve through self-care and lifestyle changes. Others require further treatment. | a. very familiar | d. heard of it | e. unclear |
| 8. Surgery is not always the next step when conservative treatments fail. For joint structure issues, surgery might help, but it is ineffective for muscle-related problems. | a. very familiar | d. heard of it | e. unclear |
| 9. Surgical treatment options for TMD include: |  |  |  |
| 9.1. Arthrocentesis: Generally performed in an outpatient setting under local anesthesia in the joint area. A puncture needle is inserted into the joint cavity, and 20–50 ml of saline solution is used for closed, pressurized repeated irrigation, or 200 ml for open continuous irrigation. | a. very familiar | d. heard of it | e. unclear |
| 9.2. Arthroscopy: A specialized small arthroscope is used to enter the temporomandibular joint, allowing the surgeon to observe its interior and perform minor procedures such as repositioning the joint disc, repairing damaged cartilage, or injecting medication under the scope. | a. very familiar | d. heard of it | e. unclear |
| 9.3 Open Surgery: An incision is made in front of the ear to expose the joint for the repair of the joint disc or ligaments. In cases of severe joint damage, more complex surgical procedures may be required to address the problem, such as bone reshaping, joint disc replacement, and in some cases, total joint replacement surgery. | a. very familiar | d. heard of it | e. unclear |

| **Part 3 Attitudes Towards Temporomandibular Joint Disorders and Their Diagnosis and Treatment** | | | | | |
| --- | --- | --- | --- | --- | --- |
| 1. I believe that temporomandibular joint disorders are a health issue that patients must pay attention to.（P） | a. strongly agree | b. agree | c. neutral | d. disagree | e. strongly disagree |
| 2. I believe that healthcare professionals should play an important role in guiding the diagnosis and treatment of temporomandibular joint disorder patients.（P） | a. strongly agree | b. agree | c. neutral | d. disagree | e. strongly disagree |
| 3. I believe that patients with temporomandibular joint disorders need further examination to determine the treatment plan. (P) | a. strongly agree | b. agree | c. neutral | d. disagree | e. strongly disagree |
| 4. I believe that education on temporomandibular joint disorders and their diagnosis and treatment should be increased for patients.（P） | a. strongly agree | b. agree | c. neutral | d. disagree | e. strongly disagree |
| 5. I believe that education and training on temporomandibular joint disorders and their diagnosis and treatment should be increased for healthcare professionals in relevant departments.（P） | a. strongly agree | b. agree | c. neutral | d. disagree | e. strongly disagree |
| 6. I believe that healthcare professionals do not pay enough attention to temporomandibular joint disorders and their diagnosis and treatment.（P） | a. strongly agree | b. agree | c. neutral | d. disagree | e. strongly disagree |

| **Part 4 Practice Regarding Temporomandibular Joint Disorders and Their Diagnosis and Treatment** | | | | | |
| --- | --- | --- | --- | --- | --- |
| 1. I actively seek to understand knowledge related to temporomandibular joint disorders.（P） | a. strongly agree | b. agree | c. neutral | d. disagree | e. strongly disagree |
| 2. I recommend that patients undergo necessary examinations to determine subsequent treatment plans.（P） | a. strongly agree | b. agree | c. neutral | d. disagree | e. strongly disagree |
| 3. I educate patients on how to recognize the symptoms of temporomandibular joint disorders and take appropriate self-management measures.（P） | a. strongly agree | b. agree | c. neutral | d. disagree | e. strongly disagree |
| 4. I guide patients in practicing relief techniques for temporomandibular joint disorders, such as heat application and relaxation techniques.（P） | a. strongly agree | b. agree | c. neutral | d. disagree | e. strongly disagree |
| 5. I provide patients with advice on the prevention of temporomandibular joint disorders.（P） | a. strongly agree | b. agree | c. neutral | d. disagree | e. strongly disagree |
| 6. I take the time to explain in detail the possible causes and treatment options for temporomandibular joint disorders.（P） | a. strongly agree | b. agree | c. neutral | d. disagree | e. strongly disagree |
| 7. I keep up with the latest literature on temporomandibular joint disorders and their diagnosis and treatment, and share and discuss it with colleagues.（P） | a. strongly agree | b. agree | c. neutral | d. disagree | e. strongly disagree |
| 8. I participate in or recommend colleagues to attend training and seminars on temporomandibular joint disorders and their diagnosis and treatment.（P） | a. strongly agree | b. agree | c. neutral | d. disagree | e. strongly disagree |
| 9. I actively participate in or promote the updating of guidelines or expert consensus on temporomandibular joint disorders and their diagnosis and treatment.（P） | a. strongly agree | b. agree | c. neutral | d. disagree | e. strongly disagree |
| 10. The sources from which you obtain information about temporomandibular joint disorders and their diagnosis and treatment are:  a. Internet consultation b. Books c. Social media d. Industry colleagues e. Searching related SCI articles | | | | | |
